# Virtual Reality for the Rehabilitation of Acquired Cognitive Disorders: A Narrative Review

**DOI:** 10.3390/bioengineering11010035

**Published:** 2023-12-28

**Authors:** Valentina Catania, Francesco Rundo, Simonetta Panerai, Raffaele Ferri

**Affiliations:** Units of Psychology I.C. and Unit of Bioinformatics and Statistics, Oasi Research Institute-IRCCS, 94018 Troina, Italy; vacatania@oasi.en.it (V.C.); frundo@oasi.en.it (F.R.); spanerai@oasi.en.it (S.P.)

**Keywords:** cognitive rehabilitation, virtual reality, serious game, videogames, attention, executive functions, language, memory

## Abstract

This review article explores the use of Virtual Reality (VR) technology in cognitive rehabilitation for individuals with neurological conditions, such as stroke, traumatic brain injury, and neurodegenerative diseases. The introduction highlights the challenges posed by cognitive impairments and the limitations of traditional rehabilitation methods. VR is presented as a transformative tool that immerses individuals in interactive environments, offering promising opportunities for enhancing cognitive functions and improving quality of life. This article covers the foundational principles of VR, its applications across different clinical conditions and cognitive domains, and evaluates empirical evidence supporting its efficacy. It also discusses the advantages, limitations, challenges, and ethical considerations in the use of VR for cognitive rehabilitation. This review concludes by exploring future developments, including advancements in VR technology, the integration of Augmented Reality (AR) and artificial intelligence (AI), and the importance of standardized assessment tools for the objective evaluation of rehabilitation outcomes.

## 1. Introduction

Cognitive impairments resulting from neurological conditions, such as stroke, traumatic brain injury, and neurodegenerative diseases, pose significant challenges to affected individuals and their families. These impairments can impact memory, attention, executive functions (EFs), spatial cognition, language abilities, and functional living skills (which progressively deteriorate as a consequence of cognitive impairments), often leading to a reduced quality of life and increased dependency. Traditional cognitive rehabilitation approaches have relied on paper-and-pencil exercises and computer-based programs, which may lack the engagement and real-world relevance necessary for effective recovery. Virtual Reality (VR) is a transformative technology that has garnered increasing attention in the field of neuroscience, neuropsychology, and cognitive rehabilitation [1,2,3,4,5,6,7]; by immersing individuals in interactive, multisensory environments [6], VR holds the promise to revolutionize the way we approach cognitive rehabilitation, offering novel opportunities for enhancing cognitive functions, promoting neural plasticity, and ultimately improving the lives of those affected by cognitive impairments [1,3,8,9,10]. Several reviews and meta-analyses have been published showing the potential benefits of VR for neuroscientific and/or neuropsychological purposes [11,12,13], and also some guidelines are starting to be available for its use [14]. This narrative review embarks on a journey through the realm of VR for cognitive rehabilitation, aiming to provide a thorough exploration of this exciting field. People suffering from acquired cognitive impairments might belong to three different clinical conditions: (1) cognitive frailty, a form of aging-related deterioration characterized by physical and cognitive impairments (particularly attention, immediate and delayed free recall, and executive functions), putting individuals at risk of Mild Cognitive Impairment (MCI), dementia, and mortality [15,16,17,18]. In this case, rehabilitation aims to restore functional skills and prevent further damage. (2) Mild Neurocognitive Disorder—mNCD (or MCI), characterized by a modest cognitive decline from a previous level of performance in one or more cognitive domains, without interference in everyday activities [19]. This condition increases the risk of developing dementia. (3) Major Neurocognitive Disorder—MNCD (or dementia), which presents with a significant cognitive decline from a previous level of performance in one or more cognitive domains, interfering with independence in everyday activities [19]. In this article, we delve into the foundational principles of VR technology, examine its applications across the three different clinical conditions and the diverse cognitive domains, evaluate the empirical evidence supporting its efficacy, discuss potential challenges and ethical considerations, and glimpse into the future of this rapidly evolving landscape. With VR technology continually advancing and its potential to offer personalized, immersive, and engaging cognitive interventions, this review aims to shed light on the current state of the art, the promises it holds, and the challenges that lie ahead in harnessing VR as a powerful tool for cognitive rehabilitation.

## 2. The Principles of Virtual Reality in Cognitive Rehabilitation

VR technology encompasses a spectrum of differently immersive experiences, from non-immersive to highly immersive environments, offering a versatile range of tools for cognitive rehabilitation [10,20]. At one end of this spectrum are non-immersive setups that typically involve standard computers, tablets, smartphones, and television screens. These setups, while less immersive, still provide valuable cognitive exercises and are accessible for a wide range of users. In this case, the individual is not totally immersed in the virtual environment; he navigates the scene in third person, but may experience both the real world, e.g., his location and the physical boundaries of the room he is working in, as well as the virtual world on the screen. Moving along the spectrum, there are more immersive tools, such as the Cave Automatic Virtual Environments (CAVEs), and headsets [21,22]; in this case, the user navigates the scene in first person. The CAVEs [23] consist of a room-sized cube with projected 3D visuals on multiple walls. However, the most common and accessible form of VR technology for cognitive rehabilitation involves head-mounted displays (HMD), such as Oculus Rift/Quest or HTC Vive. Indeed, in a CAVE, if you look down at yourself, you will see your real body; on the contrary, if you wear an oculus you cannot see your body, but you feel immersed in the virtual environment you are interacting with, knowing for sure it is not so [21]. The HMD utilize high-resolution displays and motion-tracking sensors to transport users into immersive 3D environments, but it is also possible to use less expensive devices, like Samsung Gear VR and a smartphone, to enjoy the virtual contents.

Various types of content can be used in VR to evoke different responses from both psychological and physiological perspectives. These content options range from standard and 360° photos/videos to 3D scenarios [24]. The choice of content also depends on the level of interaction with the virtual environment. For example, when using 360° videos, which are more cost-effective in terms of time and resources [18,25], the user can only change the point of view from which they observe the scene and interact with buttons specially designed for navigating the environment or receiving information. On the other hand, with 3D scenarios, users can also interact with all the enabled 3D objects within the scene.

Input devices like handheld controllers, gloves, or hand-tracking sensors allow users to interact with the virtual world, fostering sensorimotor interactions and cognitive engagement. These VR environments, whether non-immersive or highly immersive, offer a flexible and customizable approach to cognitive rehabilitation, accommodating individual needs and preferences.

Two versions of a memory task utilizing a supermarket scenario, one in full immersion with a head-mounted display and the other with low immersion on a desktop, were administered to both young adults and senior individuals. The purpose was to assess age-related differences in the use of these two platforms. The results indicated that immersive VR proved to be more fatiguing for both groups. Older adults performed better with the desktop system and reported minimal side effects. No differences were found in the preference for one platform over the other [26]. Furthermore, immersive VR was well-received by the senior participants.

VR is grounded in several fundamental principles that underpin its efficacy as a tool for cognitive rehabilitation. These principles are crucial to understanding how VR can create immersive and interactive environments that facilitate cognitive recovery.

### 2.1. Immersion and Presence

Immersion and presence are two fundamental aspects of VR that can profoundly impact the effectiveness of cognitive rehabilitation programs. Immersion is the technological capability of a system to create an illusion of reality for the user’s senses, making them an active part of the 3D environment [27]. A sensory-rich environment is provided, which fosters experiential learning. In VR for cognitive rehabilitation, immersion means that patients are not just spectators but active participants in their therapy. They can interact with virtual objects, practice real-life scenarios, and receive immediate feedback, all of which are invaluable for retraining cognitive functions. For individuals with cognitive impairments, immersion helps to bridge the gap between abstract cognitive exercises and practical, real-world challenges. For instance, a person recovering from a traumatic brain injury might use VR to navigate a virtual supermarket, practicing memory and decision-making skills in a context that closely mirrors their daily life. This kind of immersive, contextually relevant training can enhance the transfer of learned skills to real-world situations, a critical goal in cognitive rehabilitation [28,29]. A subjective correlate of immersion is the illusion of “being there”, or the sense of presence [21,30], which refers to the feeling of being physically and mentally present in the computer-generated environment, despite knowing it is not real. The greater the immersion, the greater the sense of presence [31]. Lo Priore and colleagues [32] developed the V-Store, where typical subjects could complete six sets of tasks involving categorical abstraction, programming, short-term memory, and attention. The study aimed to investigate the sense of presence in both immersive and non-immersive conditions. The results indicated a higher psychophysiological galvanic skin response in the immersive group compared to the non-immersive group.

In the context of cognitive rehabilitation, the sense of presence can be harnessed to engage patients more deeply in their therapeutic activities. When individuals feel immersed in a VR scenario, they are more likely to invest their attention and effort, leading to improved outcomes. For example, a stroke survivor participating in VR-based hand–eye coordination exercises may experience a heightened sense of presence, making them more motivated to complete their rehabilitation tasks. This increased engagement can accelerate the recovery process by encouraging consistent and enthusiastic participation in therapy.

### 2.2. Ecological Validity

Human behavior occurs within a dynamic relationship with the natural environment. Individuals do not passively perceive external stimuli, which are often multimodal and complex; instead, they actively interact with their surroundings. As a result, findings from laboratory settings may exhibit low ecological validity. Additionally, within the field of behavioral neuroscience, such findings may not accurately identify the neural mechanisms underpinning natural behavior. Virtual Reality (VR) could offer a valuable solution to this issue [33], as it allows for a high degree of experimental control while providing environments that closely resemble real-life settings [34]. It is worth noting that immersion and ecological validity are interconnected [33]. However, the ecological validity of VR for cognitive rehabilitation remains a critical point of consideration. While VR environments can be tailored to simulate real-world scenarios and challenges, the extent to which these simulations mimic the complexities of everyday life is a subject of ongoing debate. One of the key strengths of VR lies in its ability to provide controlled and repetitive exposure to stimuli and tasks, facilitating targeted cognitive training. However, the ecological validity of these exercises hinges on the accuracy of the virtual environments in replicating the challenges individuals encounter in their daily lives [35]. For instance, a VR driving simulator can help to retrain cognitive functions related to attention and decision-making, but its effectiveness relies on how closely it mirrors the unpredictability and complexity of actual road conditions. Researchers must strive to strike a balance between controlled training environments and ecologically valid scenarios to ensure that cognitive gains achieved in VR translate effectively into real-world functioning [35,36]. Moreover, assessing the ecological validity of VR-based cognitive rehabilitation necessitates a multifaceted approach. It involves not only the fidelity of the virtual environment but also the individual’s ability to generalize skills and strategies learned in VR to real-world settings. Long-term studies that track patients’ progress and functioning in their daily lives following VR rehabilitation are crucial for evaluating its ecological validity. Additionally, interdisciplinary collaboration between cognitive psychologists, VR developers, and healthcare professionals is vital to fine-tune VR applications, ensuring that they align with rehabilitation goals and the complexities of real-life challenges. While challenges remain, the continuous advancement of VR technology and research holds promise for enhancing the ecological validity of VR-based cognitive rehabilitation, offering individuals with cognitive impairments a more effective pathway toward functional recovery.

### 2.3. Embodiment and Multisensory Feedback

Embodiment and multisensory feedback play pivotal roles in the effectiveness of VR for cognitive rehabilitation. Embodiment refers to the sensation of one’s physical presence within a virtual environment [37,38]. In the context of cognitive rehabilitation, embodiment is crucial [27], as it can facilitate a profound connection between the patient and the virtual world. By embodying an avatar or virtual representation of themselves, individuals can engage in rehabilitative activities that closely mimic real-life situations. For example, a patient re-learning how to walk after a stroke can embody an avatar and experience the sensation of walking, allowing them to practice and regain motor skills in a controlled and immersive setting. This embodiment not only enhances motivation but also leverages the brain’s plasticity, potentially accelerating the recovery process [27]. Multisensory feedback is another key element in VR-based cognitive rehabilitation, and elderly people seem to benefit from multisensorial learning [39]. VR technology can provide patients with a rich array of sensory inputs, such as visual, auditory, and haptic feedback, making therapy more engaging and effective. For instance, a patient with cognitive impairments related to spatial awareness can benefit from a VR environment that provides multisensory cues about their surroundings, helping them to re-learn navigation skills. Furthermore, multisensory feedback can offer real-time information and reinforcement during therapy sessions, aiding individuals in understanding their progress and making necessary adjustments. The integration of multiple sensory modalities in VR not only enhances the overall rehabilitation experience but also provides a versatile platform to cater to a wide range of cognitive challenges.

## 3. Cognitive Domains in Rehabilitation

The following paragraphs, in this section, cover the application of VR across various cognitive domains, including attention, memory, EFs, spatial skills, and language. They also explore how VR-based interventions can adapt to different cognitive deficits, and how VR can address multiple cognitive domains simultaneously, offering a holistic approach to rehabilitation.

### 3.1. Attention

Virtual Reality has been extensively applied in attention rehabilitation for individuals with attention deficits, for example, due to stroke [40] or traumatic brain injury [41]. VR environments, reflecting real routes and landscapes, can be designed to include stimuli and distractors, mimicking real-life situations where the user’s focus is challenged. In these environments, users may be required to perform tasks while ignoring distractions and focusing on targets, gradually improving their attentional control, not only into the VR scenario, but also in real life. For example, it is possible to do an activity where users navigate (or are navigated) through a virtual path along which they name the virtual objects encountered. Another example exercise involves a VR driving simulation, where the user must maintain concentration on the road while ignoring roadside billboards or sudden roadblocks, enhancing sustained attention abilities.

There are numerous applications of VR in cognitive–motor rehabilitation, and various studies have demonstrated the significant potential and benefits, including improvements in attention, of the use of VR in patients with MCI. Training activities using games designed for the Xbox 360 Kinect have shown benefits both in the short term and in the long term [42]. Virtual reality-based physical and cognitive (dual-task) training programs have led to significant improvements in dual-task gait performance, which may be attributed to enhancements in executive function [43]. Dual-task applications have improved motivation for rehabilitation and cognitive function [44].

The use of VR has also demonstrated benefits for chronic stroke patients. For instance, it has been observed that attention, spatial awareness, and depressive mood can be positively influenced by employing an Adaptive Conjunctive Cognitive Training (ACCT) in virtual reality [45]. The utilization of the Reh@City v2.0 app, a simulator for daily living activities, has revealed improvements in various cognitive domains in everyday life [22].

### 3.2. Memory

VR is a potent tool for memory assessment and rehabilitation, addressing both short-term and long-term memory impairments. For short-term memory deficits, VR tasks might include exercises where users must remember a series of objects or locations in a virtual environment, training their working memory. Long-term memory can be targeted by immersing users in memorable virtual scenarios. For instance, individuals with Alzheimer’s disease can revisit familiar places or events from their past through VR, potentially improving autobiographical memory recall.

Virtual reality can be effectively used for both the assessment of memory impairments and memory rehabilitation. In memory assessment, the use of virtual reality can provide more comprehensive, ecologically valid, and controlled evaluations of memory than standardized tests can offer. It can also more effectively guide rehabilitation efforts tailored to the specific impairments of individual patients. Moreover, VR rehabilitation promotes procedural learning in people with memory impairments, and this improvement carries over to the real world [46,47].

While current VR rehabilitation solutions for aging and neurodegenerative diseases are still in their early stages of development, the ability to engage with body-related information, manipulate objects, receive environmental stimuli, and incorporate multisensory cues makes virtual reality one of the most promising options for spatial memory rehabilitation in aging populations [48]. Furthermore, some studies have shown that VR training, on one hand, can lead to an improvement, or at the very least, the maintenance, of cognitive functions [22,45,49]. On the other hand, it enhances motivation for rehabilitation and cognitive function [44] and is both feasible and well-tolerated by participants [50].

### 3.3. Executive Functions

VR-based interventions are particularly effective for rehabilitating EFs, such as problem-solving, planning, and decision making [51]. Users can engage in complex, real-world scenarios that demand strategic thinking. The assessment of Executive Functions (EFs) through VR applications typically involves daily-living skill tasks. Indeed, research has established a specific relationship between EFs and activities in daily living [52,53,54]. The virtual supermarket environment is the most commonly utilized, as shopping is considered an essential skill for everyday life. Supermarket and grocery scenarios [55,56,57,58,59], as well as VR versions of the Multiple Errands Test, were developed for this purpose [60,61]. Furthermore, VR environments replicating city locations, apartments [62,63,64], office scenarios [65], and kitchens for cooking tasks [66] have been created. In these scenarios, individuals must follow recipes, manage time, and make decisions about ingredient quantities, thereby exercising their planning and problem-solving abilities.

Additionally, VR-based neuropsychological tests can assess EFs in a more ecologically valid manner and may complement traditional paper-and-pencil neuropsychological assessments or enhance their psychometric validity [7,55,61,62,66,67,68,69].

Regarding the rehabilitation of executive deficits, most studies have developed scenarios based on real-life situations, in which patients perform daily living skills. A positive correlation between standard cognitive and functional measures was found in a task simulating a fire evacuation, administered to participants with MCI, mild AD, and healthy controls [64]. The use of daily living scenarios appeared to have positive effects in rehabilitating and maintaining cognitive functions in patients with stroke [70], AD [51,71,72], various types of dementia [73], and MCI [71,72,74].

Some studies have explored non-daily living virtual environments. For instance, Huang [75] combined exergaming (Fruit Ninja) with VR. Adults and older individuals (non-clinical groups) participated in a 4-week training program, showing improvements in inhibition and task switching, mediated by the sense of presence. Programs that combine VR-based physical and cognitive training have shown effectiveness not only in cognitive function but also in IADL (Instrumental Activities of Daily Living) standard measures in patients with MCI [9,76]. Another study proposed an exergame platform, the Active Brain Trainer, focused on EFs, and designed for patients with acquired brain injury (ABI) in the chronic phase. This feasibility study found both neuropsychological and functional improvements.

A recent review [11] evaluated the effectiveness of VR programs on EFs in patients with MCI. Fourteen randomized controlled trials (RCTs) were included in the study: seven of them used semi-immersive VR, four used fully immersive VR, and three used non-immersive VR. The authors suggested a positive effect of VR applications on cognitive flexibility (especially with semi-immersive and non-immersive VR), global cognitive function, attention, and short-term memory (especially with non-immersive VR) compared to the control groups.

In a recent study by Araújo et al. [77], the effects of a single session of VR, augmented reality, and neuro-functional physiotherapy on EFs and postural control in individuals with Parkinson’s disease without cognitive impairments were compared. The sessions lasted 50 min each, with a 7-day interval between them. All three intervention modalities improved both EFs and postural control.

### 3.4. Spatial Skills

Spatial cognition and navigation abilities are vital for everyday tasks, and VR can provide tailored interventions for individuals with deficits in this domain. Users can practice wayfinding in VR environments, like virtual cities or mazes, improving their spatial orientation and navigation skills. The literature on this topic was reviewed with a focus on patients presenting with various neuropsychological diseases. The results underscored the potential of navigation tasks in virtual environments to enhance navigation and orientation skills in patients with spatial memory disorders [78]. For individuals with brain injuries impacting spatial skills, exercises involving map reading, virtual treasure hunts, or even architectural design tasks can promote spatial cognition recovery. Currently, the rehabilitation of navigation ability and spatial orientation after brain damage is generally focused on training within the rehabilitation hospital or in the patient’s home as part of common physiotherapy and occupational therapy sessions.

The development of virtual reality (VR) applications may enable better generalization and the precise assessment and rehabilitation of spatial skills [79,80]. Studies with stroke patients who compared cognitive test results before and after VR training involving navigation and orientation in virtual environments have shown improvements in several cognitive domains (such as executive and visuospatial skills, language, attention, and memory skills) [22,81,82]. A controlled study with community-dwelling chronic stroke and cognitively impaired stroke patients who performed 30 min of daily exercise for 6 weeks showed significant improvements in attention, spatial awareness, and generalized cognitive functioning in the experimental group compared to the control group, who solved standard cognitive tasks at home for an equivalent period of time [45].

In the context of neurodegenerative diseases, a study was conducted with patients at risk of developing Alzheimer’s disease (AD) who performed worse than their age-matched controls, making it a potential tool for diagnosing disease development [83]. Additionally, AD patients showed impairments in VR tasks designed to study navigation skills; however, their performance could not be used to predict the degree of disorientation they might experience in the real world [84]. In one case study, a man at the onset of AD was enrolled in a cognitive treatment program based on spatial navigation in a VR environment. The results showed that the participant learned to navigate perfectly towards the desired goals in the virtual environment over the course of the training program. Furthermore, subjective feedback from his primary caregiver indicated that his ability to orient himself while driving improved significantly, and he appreciated the cognitive improvement in his daily life at home. These findings suggest that VR treatments could benefit other people with AD [85].

### 3.5. Language

VR-based language rehabilitation is beneficial for individuals with aphasia or language impairments. In VR environments, users can engage in interactive conversations with virtual characters, practicing their language comprehension and expression.

Some studies have investigated virtual reality in neurorehabilitation with the aim of analyzing its effects on specific cognitive domains, for example, memory, attention, executive functions, language, and visuospatial abilities [13,86]. The results relating to language are conflicting; in some cases, there were no significant improvements after rehabilitation based on virtual reality [13], and in other studies improvements were observed in specific areas, for example, in verbal fluency [86].

This inhomogeneity of results is confirmed by another study [87], which demonstrated a borderline-positive clinical effect of VR for the severity of the language disorder compared to conventional rehabilitation therapy, while no effects of VR were found on functional communication, word search, and on repetition.

A recent review [88] included empirical studies in which virtual reality was used to target language, well-being, or quality of life in adults with acquired language disorders. The results showed that, in general, uses of virtual reality in aphasia rehabilitation described in the literature are limited. Most applications target the repair of speech impairments, and the identified studies used known published protocols delivered through the new VR format.

Giachero et al. [89] conducted a study in which thirty-six people with chronic aphasia (PWA) were randomly assigned to two groups. The VR group underwent conversational therapy while observing daily life in VR, while the control group was trained in a conventional environment without VR support. Within-group comparisons showed significant improvement in several language tasks only in the VR group. Significant gains, after the treatment, were also found in the VR group in various psychological dimensions, for example, self-esteem and emotional and mood state.

In a quasi-randomized study on a group of people with aphasia, a platform called Eva Park was tested, which contained a number of functional and fantastic places and allowed for interactive communication between multiple users. After a 5-week training program, significant improvements in functional communication were noted; there was excellent compliance with the intervention, with no participant lost to follow-up [90].

Marshall et al. [91] investigated the possibility of providing group social support to people with aphasia via a multi-user virtual reality platform with the aim of promoting well-being and communication success. The feasibility results showed that the recruitment objective was achieved with excellent participant compliance, while no significant change was observed in any of the outcome measures (well-being, communication, social connection and quality of life). Overall, however, the data suggested that a broader trial of remote group support, using virtual reality, would be worthwhile.

Regarding the use of VR in the assessment of acquired language disorders, the study by Wall et al. [92] shows preliminary evidence that the VR cognitive assessment app for aphasia is a feasible cognitive assessment for stroke survivors with and without aphasia.

There are currently no VR applications that have been designed to assess or treat cognitive-communication disorders (CCDs) following traumatic brain injury (TBI). A study of Brassel et al. aimed to explore the views of speech–language pathologists (SLPs) who work with people who have a TBI to generate ideas and considerations for using VR in rehabilitation for CCDs [93]. Useful suggestions emerged from the thematic analysis to overcome possible obstacles to the use of VR, and the idea also emerged that VR could be a very useful tool for improving clinical practice.

These interactions can be customized to target specific language deficits, such as naming difficulties. Furthermore, VR environments can simulate real-life scenarios like a grocery store or a restaurant, enabling users to practice functional language skills in context.

### 3.6. The Activities of Daily Living and the Instrumental Activities of Daily Living

VR-based programs have emerged as groundbreaking approaches for improving the activities of daily living (ADL) and the instrumental activities of daily living (IADL) [56,74,94], especially for individuals with various mental disorders such as MCI and AD [72]. These applications can be personalized according to the strengths and weaknesses of the patients, immersing them in realistic virtual environments where they can practice a wide range of tasks, from basic self-care routines to more complex activities like grocery shopping or managing finances. VR therapy not only enhances physical and cognitive skills but also fosters a sense of independence and confidence in individuals striving to regain control over their daily lives.

Only a few studies have developed programs with scenarios for daily living to re-learn specific functional living skills and evaluate the impact that this re-learning has on actual skills in natural environments [28,29,71,95,96,97,98,99]. The results of these studies will be described in Section 4. However, some general indications can be drawn:

1. The utility of familiarization training before starting real VR training, especially for older people who are less familiar with even simple technological devices.
2. Virtual training (VT) should have a sufficient duration, of at least a few weeks, to allow for the re-learning of functional living skills.
3. The development of the virtual application should include not only feedback but also error corrections and prompts to help patients to produce the correct responses.
4. VT variable scores and/or standard neuropsychological and quality of life measures should be included, as well as questionnaires on agreement, acceptability, and negative side effects.

In the virtual assessment of everyday functions, a study by Allain et al. [100] used a non-immersive virtual coffee machine for a coffee-making task with patients with AD. The authors aimed to compare virtual and real tasks, as well as to find links between the virtual task and global cognition, executive functions, and IADL (as reported by caregivers). The findings showed correlations between the virtual task and all the cognitive and IADL measures, as well as the ability of the virtual task to predict the actual skill.

Another recent study aimed to validate an immersive VR neuropsychological battery (VR-EAL) [101]. The correlation with the corresponding paper-and-pencil battery was statistically significant. However, the advantages of the VR-EAL were the shorter time of administration, its ecological validity, pleasantness as judged by participants, and the absence of cybersickness.

### 3.7. A Holistic Approach

VR-based interventions excel in addressing multiple cognitive domains simultaneously. For example, a VR-based shopping scenario can require users to plan their shopping list (EFs), navigate through the store (spatial skills), maintain attention to the task (attention), recall item names (memory), and engage in conversations with store attendants (language). This holistic approach is particularly valuable for individuals with complex cognitive deficits, promoting comprehensive cognitive rehabilitation. VR technology’s adaptability and versatility make it a valuable tool for creating tailored interventions that cater to the specific cognitive needs of each individual, fostering a more inclusive and effective approach to cognitive rehabilitation.

### 3.8. Stress and Cognitive Load

The intersection of VR and human psychology has also been explored. Kim et al. [102] analyzed the stress-alleviating potential of VR by conducting an open randomized crossover trial on individuals experiencing high stress levels. Their findings reveal that exposure to VR not only significantly reduces stress but also induces positive changes in physiological parameters, particularly heart rate variability. The study suggests that immersive VR experiences have promising applications in stress management interventions. Complementing this, Collins et al. [103] contributed to the discourse by proposing a methodology to measure cognitive load and insight in VR learning contexts. By employing a mixed-methods approach, combining self-reporting measures and physiological data, they offer a comprehensive understanding of cognitive processes in VR. This integrated approach sheds light on the intricate relationship between stress reduction, emotional load, and cognitive processes in VR environments, paving the way for more refined and effective applications in areas such as stress management and virtual learning experiences.

### 3.9. Multisensory Feedback

Multisensory feedback, particularly in the context of integrating VR with biofeedback mechanisms such as electroencephalogram (EEG) and electrocardiogram (ECG), represents a groundbreaking approach to enhance user experience and therapeutic interventions. Studies exploring the synergy between VR and biofeedback have demonstrated promising outcomes in various fields [104]. For instance, in the realm of rehabilitation, VR coupled with biofeedback can facilitate motor skill development and cognitive rehabilitation by providing immersive and responsive environments [105]. The integration of EEG and ECG data into VR experiences not only enhances the realism and interactivity of virtual environments but also provides valuable insights into users’ cognitive and emotional states. This holistic approach to feedback systems not only elevates the immersive quality of VR but also holds great potential for advancing fields such as healthcare, education, and mental well-being.

## 4. The Advantages and Limitations of VR-Based Cognitive Rehabilitation

### 4.1. The Advantages of VR-Based Cognitive Rehabilitation

VR offers several distinct advantages for cognitive rehabilitation that can significantly benefit individuals with cognitive impairments. Firstly, VR provides a controlled and customizable environment, allowing therapists to design rehabilitation programs tailored to the specific needs and abilities of each patient. This personalization ensures that cognitive exercises are challenging yet achievable, fostering a sense of accomplishment and motivation. For instance, a person recovering from a traumatic brain injury can engage in VR-based cognitive tasks like memory games, gradually increasing the difficulty level as their cognitive abilities improve. This adaptability not only makes therapy more effective but also reduces frustration, making patients more likely to stay committed to their rehabilitation regimen.

Secondly, VR promotes real-world relevance and the transfer of skills. Traditional cognitive exercises often take place in clinical or laboratory settings and may struggle to bridge the gap between rehabilitation and everyday life. In contrast, VR can simulate real-life scenarios, allowing patients to practice cognitive skills in context. For example, a stroke survivor can use VR to navigate a virtual supermarket, re-learning decision-making, memory, and spatial orientation skills that directly apply to their daily activities. This contextual learning enhances the transfer of cognitive gains from therapy to real-world situations, ultimately improving the individual’s overall quality of life and independence. The immersive nature of VR enhances engagement and encourages patients to apply newly acquired skills outside of the therapy sessions, making it a powerful tool for cognitive rehabilitation.

### 4.2. The Limitations of VR-Based Cognitive Rehabilitation

While VR offers promising avenues for cognitive rehabilitation, it is essential to acknowledge its limitations. Firstly, the cost and accessibility of VR technology can pose significant barriers. High-quality VR systems often require substantial financial investments, making them less accessible to many individuals and healthcare facilities. This limitation could result in unequal access to potentially beneficial cognitive rehabilitation tools, creating disparities in the quality of care. Additionally, VR systems can be physically challenging for some patients, particularly those with severe motor impairments or sensory deficits. Customizing VR experiences to accommodate a wide range of physical and cognitive abilities is a complex endeavor, and it may not be feasible for all patients.

Moreover, the long-term effectiveness and durability of cognitive gains achieved through VR-based rehabilitation require further investigation. While initial studies show promise, more research is needed to assess whether the cognitive improvements observed within VR environments translate into meaningful and lasting real-world outcomes. Additionally, not all cognitive impairments may be equally amenable by VR-based interventions, and the technology may not be suitable for every individual. Careful patient selection and ongoing assessment are necessary to ensure that VR is the right fit for a particular rehabilitation program. Furthermore, the potential for cybersickness or discomfort within VR experiences can limit their utility, especially for older adults or those with preexisting medical conditions. These limitations emphasize the importance of a thoughtful and individualized approach when considering VR for cognitive rehabilitation.

One significant challenge faced in VR-based cognitive rehabilitation is the occurrence of cybersickness, a phenomenon akin to motion sickness experienced within virtual environments [39,106]. Cybersickness encompasses symptoms such as nausea, dizziness, and discomfort, which can arise due to the disparity between visual and vestibular sensory inputs. For individuals engaged in cognitive rehabilitation, particularly those with pre-existing neurological conditions, cybersickness poses a hindrance to the seamless integration of VR technology into therapeutic interventions. The immersive nature of VR, while offering potential benefits, can lead to adverse effects on user experience, potentially causing discomfort and reluctance to participate fully in rehabilitation sessions. Mitigating cybersickness involves addressing factors such as latency, display quality, and motion dynamics within VR environments [107]. Striking a balance between the immersive qualities of VR and minimizing adverse effects is crucial for ensuring the acceptability and effectiveness of VR-based cognitive rehabilitation programs. As the field evolves, researchers and developers must continue to explore strategies to reduce cybersickness, enhancing the overall accessibility and feasibility of VR interventions for individuals undergoing cognitive rehabilitation.

## 5. The Empirical Evidence Supporting VR-Based Cognitive Rehabilitation

### 5.1. The General Impact of VR Interventions

The empirical evidence supporting the use of VR in cognitive rehabilitation has been steadily growing, highlighting its potential to enhance cognitive functions and quality of life for individuals with various neurological conditions [6,108,109,110,111]. Numerous studies have demonstrated the effectiveness of VR interventions in improving well-being [4,112,113], symptoms of depression, apathy, anxiety, and agitation [45,112,114,115], perceived stress [116], global cognition, and specific cognitive domains such as attention, memory, executive functions (EFs), and spatial awareness [6,110,116].

### 5.2. Specific Cognitive Domains

In a randomized controlled trial (RCT) by Thapa et al. [117], a full-immersive VR rehabilitation program, including four games (juice making, crow shooting, fireworks, and love house), was carried out with patients with Mild Cognitive Impairment (MCI) for 24 sessions (three times per week), resulting in improved executive and physical functions. Two studies by Park et al. [44,50] used, respectively, non-immersive and full-immersive VR cognitive training with older adults with MCI. No cognitive improvements were found after the non-immersive VR cognitive training (attention, memory, problem solving, and EFs) [50]; however, the program turned out to be feasible and tolerable for participants with MCI. On the contrary, after the full immersive VR program (30 min per day, 5 days per week, for 6 weeks), improvements in general cognitive functions, divided attention, and short-term memory were found, as well as interest and motivation higher than in the control group, who carried out traditional rehabilitation. A 360 Kinect cognitive game [42] was used for evaluating the short- (one session) and long-term (six-week sessions) effects of training on cognition, slowness, and EEG complexity in MCI. Only in the long-term condition were some improvements found in global cognition and attention. Combined cognitive and physical training was developed by Liao et al. [43], aiming to evaluate its effects on EFs and dual-gait performance (both motor and cognitive dual-task), as well as to compare it with a traditional approach. The program included 36 sessions over 12 weeks, administered to older adults with MCI. Only the VR-trained group showed better performance in cognitive dual-task gait (walking while performing serial subtraction); both groups showed improvements in EFs as measured with the Stroop test, as well as in single task and motor dual-task gait (walking while carrying a tray). Some improvements in EFs, memory, and verbal fluency measures were found in a small MCI group trained with a VR program combining aerobic and cognitive tasks [86]; however, no statistically significant differences were found when compared with non-trained controls. Worse performances were found in tests on the activities of daily living, but participants instead showed a perception of improvement in the questionnaires. These results may probably be due to the small number of participants in the study (ten in total). Also, Hughes et al. [118] found no statistically significant differences between Wii interactive video gaming and health education groups, despite a medium effect size in favor of the Wii group with MCI, both in cognitive and physical functioning.

Research has shown that stroke survivors can benefit from VR-based cognitive rehabilitation exercises that mimic real-world tasks, resulting in significant improvements in cognitive performance. The non-immersive Adaptive Conjunctive Cognitive Training by Maier et al. [45] was administered to chronic stroke patients with MCI for six weeks, 30 min a day, resulting in improved attention, spatial awareness, and general cognitive function; on the contrary, no gains were found in EFs and memory. Furthermore, participants showed decreased depressive symptoms, but they returned to the pre-training levels at follow-ups. Instead, controls showed no change over time. A virtual simulation of a city (Reh@City) was developed by Faria et al. [22]: memory, attention, visuo-spatial abilities, and EF tasks were integrated into several daily routines. Nine stroke patients were trained with Reh@City, and another nine with traditional rehabilitation. Statistically significant improvements were found in global cognitive functioning, attention, and EFs when comparing the VR-trained group to the conventional therapy group, as measured with a neuropsychological battery administered pre- and post-training.

An immersive VR reminiscence intervention [119] was carried out with patients with dementia in order to evaluate its immediate and prolonged (3–6 months after) effects on cognition, depressive symptoms, global status, and caregiver burden. The VR therapy lasted 3 months, including two sessions per week. The authors found a reduction in depressive symptomatology immediately after the end of the training. Cognitive functions showed a worsening at follow-ups, compared with performances at the end of the training, thus suggesting that reminiscence VR training might play a significant role in preserving cognitive skills. In a pilot RCT by Oliveira et al. [51], an improvement in overall cognitive function was found in patients with mild-to-moderate Alzheimer’s disease (AD) compared with controls after a virtual-based cognitive intervention (two sessions per week for a total of 10 sessions over two months), reproducing several Instrumental Activities of Daily Living (IADL). This protocol has also been shown to be effective in maintaining cognitive skills in people with AD. With a Chinese supermarket immersive virtual environment, more improvements were found in participants with AD than in those with MCI in global cognitive functioning, memory, attention, and EF [116].

Instead, no improvements were found in the cognitive skills of people with dementia after a GRADYS game intervention, but only in participants without dementia [120]. Fasilis et al. [73] evaluated the effectiveness of an Interactive Computer Training–Serious Game on cognitive functions. They developed three daily living tasks (shopping from the supermarket, preparing breakfast, and tidying up and cleaning the house), each of them including three levels of increasing difficulty. Patients with various types of dementia used the game for 4–5 weeks, for a total of 10 h. The study included the familiarization and training phases, as well as pre-, during-, and post-intervention assessments with a broad neuropsychological battery. After training, increased scores in some memory (story recall) and general executive functions were found; on the contrary, no improvements were found in working memory, attention, and problem solving.

### 5.3. Studies on Mild Cognitive Impairment

Few studies in the literature applied VR to the cognitive rehabilitation of people with cognitive frailty. A virtual reality (VR) simultaneous motor–cognitive training program, including cycling on an ergometer and cognitive games, was carried out over 8 weeks with nine patients with cognitive frailty [106]. The intervention group showed a significantly larger improvement in cognitive function than the control group (who carried out motor and cognitive rehabilitation on a non-VR platform); instead, the reduction in physical frailty was similar in both groups. A Brain m-App, designed for the home-based rehabilitation of spatial memory, attention, and EF, was presented by Pedroli et al. [18] in a clinical study with 10 older frail participants. This application used an innovative tablet device and was based on the 360_videos technology; the training lasted 10 days and included cognitive exercises. The results showed improvements in EF and spatial memory performances. This approach seems to be a valid option to continue at home the rehabilitation that began at the hospital. The device was found to be easily usable by people with cognitive frailty, even if complete independence was not achieved. A previous study by the same research team [121] aimed to evaluate the effects of a VR system, Positive Bike, on the cognitive and motor performances of frail people, on the basis of the dual-task paradigm. The system was made by a cycle ergometer connected with a CAVE. Good usability and an enjoyable user experience were found. A Nintendo Wii Fit Plus interactive video game was used to evaluate its acceptability, safety, and feasibility by participants in the frailty and pre-frailty conditions [122]. Fourteen training sessions were performed, twice a week, and three assessments were carried out (at pre- and post-training, and at a follow-up 30 days after the end of the intervention). This program was found to be feasible, acceptable, and safe; its administration produced motor improvements, but no improvements were found in cognitive performances.

### 5.4. Studies on Functional Living Skills

Finally, let us take a look at the studies on the rehabilitation of functional living skills. Hoffmann et al. [95] developed an interactive computer-based cognitive program with individuals with AD. The task consisted of finding a shopping route and buying three items, then answering some questions about the experience. Some parameter scores were collected (number of errors, time to buy the items, number of correct answers, and instruction repetition). AD patients performed worse than the control groups (healthy controls and individuals with depression); however, their task performances significantly improved. Van Schaick et al. [96] investigated both facilitators and barriers for a walk in a town center in people with dementia. Participants were asked to walk in both real and virtual conditions. Authors found performance improvements and their generalizability from virtual to real-world scenarios. Foloppe et al. [97] used VR combined with errorless learning and a vanishing-cue in a single case study with a patient with AD, in order to promote autonomy in cooking activities, finding improvements in performances and stability over time, as well as the transfer of skills to real life. Two other cooking tasks (preparing toast and a cup of coffee) were proposed by Yamaguchi et al. [98], in which two error-reduction-learning methods were tested (vocal or written instruction). Patients with AD performed worse than controls at the first assessment, with a greater number of errors, perseverations, and omissions. However, after a short learning session, performances similar to those of controls were achieved, with decreased perseverations and the number of vocal and written prompts needed to produce the correct response. Manera et al. [71] developed a tablet-based “kitchen and cooking” game to evaluate its acceptability for patients with MCI and AD. Participants were asked to use the game for as long as they wanted over the course of 4 weeks, and once a week, data on acceptability, motivation, and emotions were collected via self-report questionnaires, as well as objective data such as time spent playing, and the number of errors. After one week, improvements in practical and cognitive functions were found. The authors concluded that they were in favor of good acceptability of the game for both patients with MCI and AD, and confirmed its usefulness for the rehabilitation of functional living skills. Furthermore, the game seemed to work even in patients with symptoms of apathy.

Our research team carried out first a feasibility study [99] and two clinical studies [28,29] on the effectiveness of non-immersive VR training on functional living skills as well as the possible transfer to the real-life application of the re-learned skills, in patients with Major Neurocognitive Disorder (M-NCD) due to different etiologies, with a prevalence of Vascular and Alzheimer’s dementia. A digital system was set up, including a server connecting the database to the suite of apps to provide information (taking medicines, preparing a suitcase, and shopping at the supermarket), which was installed on the touch TV.

Some principles and procedures of Applied Behavior Analysis were used for developing the apps, including verbal reinforcements, correction procedures, and task analysis. Written and vocal instructions were provided. In vivo tests were carried out before (at T1) and after the training (at T3). A familiarization phase was added in the clinical studies. Data were collected related to virtual tasks (total execution time, number of errors, number of clues, and omitted responses) and in vivo test parameters (total execution time and number of errors). The results of a feasibility study [99], in which patients performed 10 VR sessions for each task, showed that such VR training could be feasible for the rehabilitation of functional living skills in patients with mild-to-moderate dementia. An improved performance was observed, both in VR training and in in vivo tests, with a spontaneous transfer of the re-learned skills to natural environments. The control group (administered traditional cognitive stimulation training) showed no improvements in the in vivo test, suggesting the need for specific training for each functional living skill. The results suggested the possibility of teaching IADL in a safe and controlled environment, adapting tasks to individuals’ characteristics, enriching traditional cognitive rehabilitation, expanding the use of VR training at home without a trainer for the continuation of treatment begun at the hospital, and collecting accurate and complete data. Furthermore, participants enjoyed using the tool.

The first clinical study [28] included a broader sample (24 and 18 participants, respectively, in the experimental and control groups). Participants in the experimental group were administered 10 to 20 sessions of VR training. In vivo tests were carried out at T1 and T3 with both groups. The results confirmed those of the feasibility study; as for VR training, execution times and the number of clues decreased, while correct responses significantly increased. Comparisons between the experimental group and controls showed statistically significant differences in the in vivo tests, where controls showed no improvements. Participant satisfaction was moderate to high. A qualitative and quantitative analysis was also conducted by comparing the first session with each of the following sessions to elucidate how our VR training worked. The most significant changes occurred within the first 7–10 sessions, with some minor changes afterward, indicating, in our opinion, that a good format for obtaining the best outcomes and preventing fatigue and boredom might include cycles of 10 sessions each, interspersed with break times.

Finally, the second clinical study [29] aimed to compare the effectiveness of VR training for participants with M-NCD due to neurodegenerative and non-neurodegenerative conditions. Ten sessions of VR training were administered for each task. Both groups significantly improved in all variable scores, both in the VR training session and in the in vivo tests, with better outcomes in the non-neurodegenerative condition; however, no statistically significant differences were found between them. On the contrary, differences were found in the comparisons with the respective control groups in the in vivo tests. A moderate-to-high satisfaction was found, as well as no problems or adverse events while using the tool. The ecological validity of our system seemed to be verified, due to the improvements in the trained daily skills not only in the virtual environment but also in the natural one in the early stages of dementia.

### 5.5. Considerations and Future Directions of VR Systems

As a conclusion to this section, we can make some considerations: the results of the studies above seem to suggest that VR-based programs for individuals with acquired cognitive disorders are valid for improving or maintaining cognitive functions, re-learning daily living skills, and improving patients’ involvement and adherence to rehabilitation. VR seems to be feasible, tolerable, acceptable, enjoyable, and motivating. This is particularly important in long-term rehabilitation where maintaining interest and compliance can be challenging for people with AD, fronto-temporal dementia, MCI, and frail elders, who seem to prefer it to traditional cognitive rehabilitation. Furthermore, it appears to produce better outcomes than traditional rehabilitation or no treatment. The ability to track progress in real-time and receive immediate feedback within VR environments also contributes to better adherence and, ultimately, more substantial cognitive gains. These findings underscore the capacity of VR to provide tailored, engaging, and task-specific therapy that traditional methods may struggle to replicate.

Positive outcomes were also described in reducing symptoms of anxiety, depression, apathy, and agitation, as well as in improving coping strategies, perceived stress, well-being, and quality of life. Concerning cognitive rehabilitation, the versatility of VR allows for a wide range of cognitive exercises and simulations tailored to an individual’s specific needs and goals. Researchers and clinicians can create and adapt VR environments to challenge patients at their current cognitive level, gradually increasing the difficulty as they progress. This adaptability has been particularly beneficial for individuals with conditions like AD, where cognitive decline varies from person to person. Most studies showed an improvement in general cognition; however, in some studies, no improvements were found in participants with dementia, and some authors concluded that VR is more useful in preserving cognitive functions than in improving them. Improvements in specific cognitive functions such as EF, attention, and memory were not always found after VR training. The discrepancies between the results of some studies might be due to differences in purposes, devices used, programs, and protocols (in terms of times, the number and length of sessions, procedures used, the presence or absence of the trainer, and so on).

Semi-immersive VR was considered in some studies to be more effective than fully immersive VR in improving cognitive performances in AD and MCI, and it might be more easily repurposed for continuing the rehabilitation activities at home since it involves the use of a technological tool that may be managed independently by patients, without the constant presence of the trainer.

Despite the benefits of using VR for cognitive stimulation, some critical points remain to be solved and further investigated. For example, the comparison between VR and real-world performances to confirm the effectiveness of VR-based programs compared with traditional interventions in hospitals or at home. The spontaneous transfer of skills performed in VR laboratories to the natural environment must also be explored in depth, as this is a key point to establish the ecological validity of VR training. Increasing self-perception, self-esteem, and self-confidence, as well as the duration of the positive outcomes over time, also need further examination, as most studies presented short-term results. The sickness symptoms of some immersive technological tools must also be addressed and solved.

Finally, based on the studies available to date, it would be appropriate to begin drawing up practical guidelines for the development and administration of VR-based interventions in people with acquired cognitive and motor disorders. To date, VR is not very widespread in healthcare services, and some barriers, such as the complexity of the technical setup, as well as the difficulties and the prolonged times required for the development of VR applications, contribute to slowing down their use in caring for people with motor and cognitive deficits. As the body of empirical evidence continues to grow, VR-based cognitive rehabilitation is increasingly recognized as a valuable and innovative tool in the arsenal of therapies aimed at improving cognitive function and overall quality of life for individuals with cognitive impairments. However, a simplification of the virtual technological systems is needed, as VR interventions could significantly improve the quality of healthcare and produce savings on rehabilitation for healthcare services and families.

## 6. Challenges and Ethical Considerations

The widespread adoption of VR in cognitive rehabilitation, while promising, poses some challenges. Accessibility remains a key concern, with VR systems often requiring a level of technical proficiency and physical capability that may limit access for individuals with severe cognitive or physical impairments. Additionally, the cost of VR technology, including headsets and software, can be prohibitive, potentially excluding those with limited financial resources from its benefits. Usability issues, such as the complexity of VR interfaces and the need for training, can pose obstacles, particularly for older adults or individuals with cognitive deficits. Moreover, VR interventions may lead to potential adverse effects, including motion sickness, simulator sickness, or discomfort, which could deter some individuals from participating fully in therapy. As the field of VR-based cognitive rehabilitation continues to evolve, addressing these accessibility, cost, usability, and safety considerations will be crucial to ensuring equitable access to its promising cognitive benefits.

In the realm of VR-based rehabilitation, ethical considerations surrounding privacy, informed consent, and patient autonomy come to the forefront. Firstly, as VR systems often collect detailed user data and personal information for treatment and assessment purposes, preserving patient privacy becomes paramount. Safeguarding sensitive data from breaches or unauthorized access is essential to maintain patient trust and confidentiality. Secondly, informed consent takes on a unique dimension in VR, as patients must not only understand the nature of their therapy but also the potential for data collection and its implications. Patients must be provided with clear and transparent information about how their data will be used, stored, and shared to make informed decisions about their participation. Lastly, ensuring patient autonomy in VR-based rehabilitation means affording individuals the freedom to make choices about their treatment plans and the nature of their virtual experiences. Patients should have agency in selecting and modifying VR exercises to align with their preferences and needs, empowering them in their rehabilitation journey while respecting their individual autonomy and values.

## 7. Future Developments and Conclusions

The future of VR-based cognitive rehabilitation holds exciting possibilities that can significantly enhance the field. Firstly, advancements in VR technology are expected to lead to more accessible and cost-effective systems, making cognitive rehabilitation using VR more widely available. These technological innovations may include lighter and more comfortable headsets, improved motion tracking, and enhanced haptic feedback, providing a more immersive and user-friendly experience. Additionally, the integration of Augmented Reality (AR) into cognitive rehabilitation can enable real-time assistance and feedback in the user’s physical environment, further blurring the lines between virtual and real-world rehabilitation.

Moreover, the incorporation of artificial intelligence (AI) into VR-based cognitive rehabilitation is on the horizon. AI algorithms can customize interventions based on real-time user performance and adapt to individual progress, offering tailored exercises that maximize cognitive gains. Natural language processing and speech recognition AI can facilitate language therapy, while machine-learning models can analyze user data to provide therapists with actionable insights, streamlining treatment planning. Furthermore, AI can contribute to the gamification of cognitive exercises, making them more engaging and enjoyable for users. To harness the full potential of VR-based cognitive rehabilitation, standardized assessment tools that are specifically designed for VR environments are essential. These tools should measure progress across cognitive domains effectively, allowing for objective and comparable evaluations of rehabilitation outcomes. By developing standardized assessments that align with the immersive and dynamic nature of VR, researchers and clinicians can more accurately track cognitive improvements, enabling evidence-based interventions and enhancing the credibility of VR-based cognitive rehabilitation as a therapeutic approach.

## Data Availability

No new data were created or analyzed in this study. Data sharing is not applicable to this article.
